# Focal adhesion kinase as a new player in the biology of onco-hematological diseases: the starting evidence

**DOI:** 10.3389/fonc.2024.1446723

**Published:** 2024-08-30

**Authors:** Guido Capasso, Nayla Mouawad, Maria Castronuovo, Edoardo Ruggeri, Andrea Visentin, Livio Trentin, Federica Frezzato

**Affiliations:** Hematology Unit, Department of Medicine, University of Padova, Padova, Italy

**Keywords:** FAK, microenvironment, tumor, blood, hematological diseases

## Abstract

Focal adhesion kinase (FAK) is a non-receptor tyrosine kinase mainly found in the focal adhesion regions of the plasma membrane and it has a crucial role in migration and the remodeling of cellular morphology. FAK is also linked to several aspects of cancer biology, from cytokine production to angiogenesis, drug resistance, invasion, and metastasis, as well as epithelial-to-mesenchymal transition. The gene locus of FAK is frequently amplified in several human tumors, thus causing FAK overexpression in several cancers. Furthermore, FAK can influence extracellular matrix production and exosome secretion through cancer-associated fibroblasts, thus it has an important role in tumor microenvironment regulation. Although the role of FAK in solid tumors is well known, its importance in onco-hematological diseases remains poorly explored. This review collects studies related to FAK significance in onco-hematological diseases and their microenvironments. Overall, the importance of FAK in blood tumors is increasingly evident, but further research is required to confirm it as a new therapeutic target in hematological contexts.

## 1 Introduction

Focal adhesion kinase (FAK) is an intracellular protein of 125 kDa (1) involved in several biological processes, such as cellular modeling, adhesion, and motility (2). FAK is usually located on the cytosolic side of the plasma membrane, in regions called “focal adhesions (FA)”, which are rich with surface receptors, such as integrins (2).

During the inflammatory process and antigen presentation as well as target-cell recognition, migration and motility are crucial for leukocytes, the cell type covered in this review. For all these processes, leukocyte polarization is required and there is evidence that suggests that FAK is located on and acts mainly on the leading edge of the migrating immune cells; it has been shown that FAK activity occurs in concert with integrins (3, 4). FAK has been studied more in cell types of solid tissue origin than in leukocytes; for this reason, further research about FAK in the hematological/immunological context is required, and the aim of this review is to shed light on the current knowledge of FAK within the immune cell, with a particular focus on hematological malignancies.

**Figure 1:**
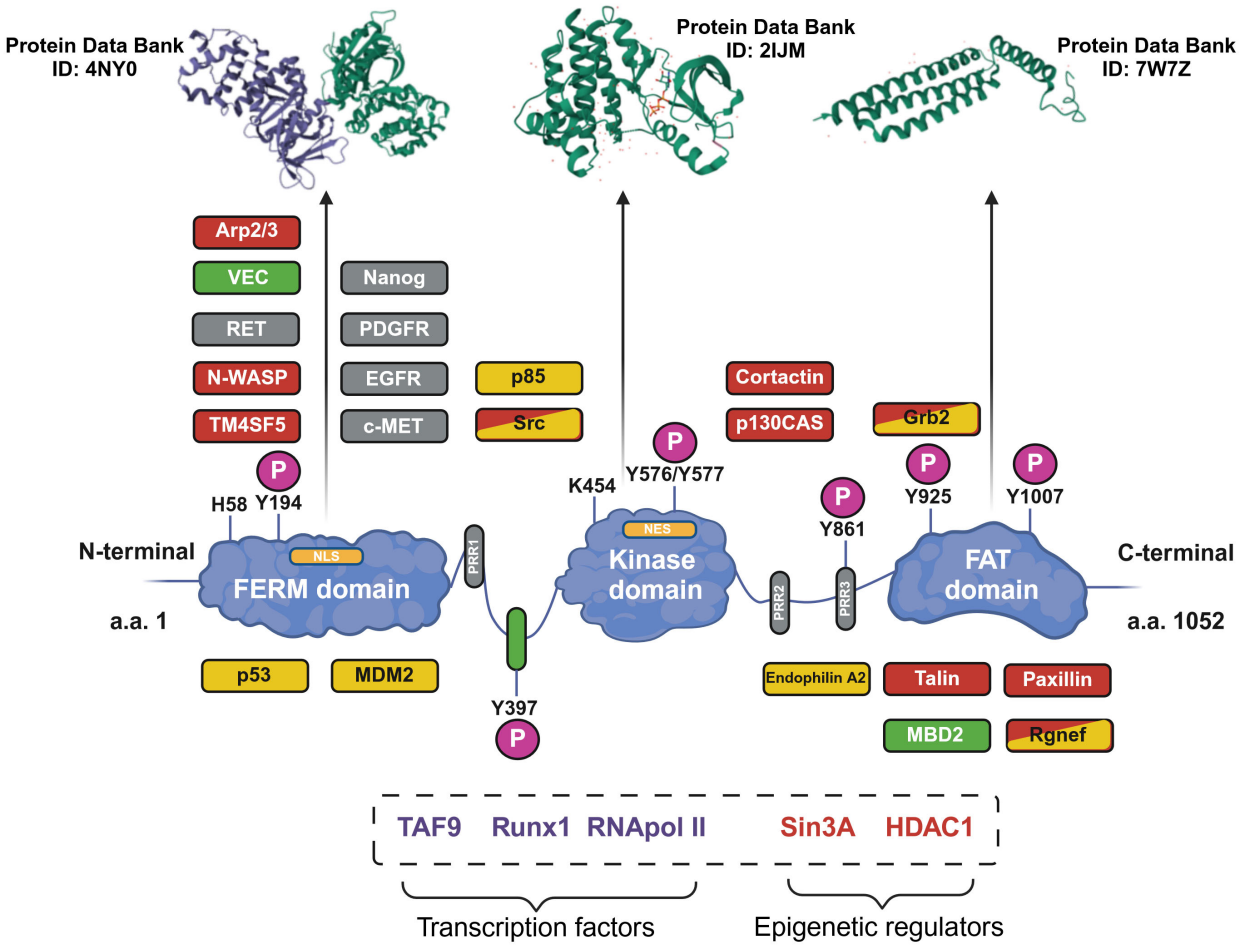
Structure of the focal adhesion kinase (FAK) protein. The structure of FAK is shown with the three main domains. The phosphorylation sites (P) and proline-rich regions (PRRs) are shown. The main protein interactors of FAK are depicted in the zones corresponding to the binding site; interaction partners are related to cell motility (orange), cell survival (yellow) or to both functions (orange and yellow). Additionally, proteins related to FAK activation (gray) and the tumor microenvironment (green) are shown. In the dotted box, other FAK interaction partners, for which the exact binding sites remain uncharacterized, are shown. Furthermore, the tri-dimensional structures of the three main domains of FAK are shown in the upper part of the image (the Protein Data Bank database ID number is reported for each domain). Image created with Biorender.com (under the license of the University of Padua).

At a structural level (**Figure 1**), FAK is composed of three domains: the N-terminal FERM domain (4.1-ezrin-radixin-moesin), a central domain provided with kinase activity, and the C-terminus FAT domain containing the focal adhesion targeting (FAT) sequence responsible for FAK localization at the focal adhesions (5). These three domains are connected by two linker portions containing proline-rich regions (PRRs) (5). The FERM domain contains a nuclear localization sequence (NLS) and the kinase domain contains a nuclear export sequence (NES), which enable FAK to shuttle between the cytosol and the nucleus (6). Furthermore, phosphorylation sites and binding sites for protein partners are disseminated along FAK structure (5).

FAK performs signaling and scaffolding functions and is activated by several receptors, such as integrins and growth factors receptors, e.g., epidermal growth factor receptor (EGFR) and platelet-derived growth factor receptor (PDGFR) (2). A fundamental step in FAK activation is its autophosphorylation (p) at the tyrosine (Y) 397 residue (pFAKY397); this creates a motif recognizable by SH2-domain-containing proteins, such as Src-family kinases, which further phosphorylate FAK and complete its activation process (2). FAK has been correlated with several pro-tumorigenic aspects, such as the induction of immunosuppressive cytokines, promotion of angiogenesis, support of self-renewal capability, drug resistance, invasiveness and metastasis, and epithelial-to-mesenchymal transition (7, 8). In addition, owing to amplification processes of the encoding *PTK2* gene (on chromosome 8, in humans), FAK is overexpressed in several human tumors, such as gastric cancer and colorectal cancer (2, 5). Eventually, FAK is also a key player in the solid tumor microenvironment, for instance increasing extracellular matrix production and exosome secretion by cancer-associated fibroblasts (6).

There is a lot of evidence about the role of FAK in solid tumors in the literature (9–17), which has led to the development of clinical trials in which inhibitors of FAK, such as defactinib, were tested in solid cancers with promising results (5). Despite this, FAK remains little investigated in blood tumors (18), especially from a clinical point of view, with only a few ongoing trials in which FAK inhibitors (e.g., defactinib) are being tested (**Table 1**). This last aspect reinforces the necessity to study FAK in the context of blood tumors. With these premises, we herein harvested the main evidence about the role of FAK in blood neoplasms and in their microenvironment, thus representing a starting point for the development of further studies in this field.

**Table 1:** Clinical trials related to the use of the main FAK inhibitors in hematological malignancies.

FAK inhibitor	ClinicalTrials.gov ID number	Title of the study	Status	Conditions	Phase	First posted in
Defactinib (VS-6063)	NCT05636514	Combined Evaluation of Epigenetic and Sensitizing Therapy in AML and MDS	recruiting	▪ Myelodysplastic syndromes ▪ Chronic myelomonocytic leukemia ▪ Acute myeloid leukemia	1	2022
Defactinib (VS-6063)	NCT04439331	Testing VS-6063 (Defactinib) as a Potential Targeted Treatment in Cancers With NF2 Genetic Changes (MATCH-Subprotocol U)	Active, not recruiting	▪ Advanced lymphoma ▪ Advanced malignant solid neoplasm ▪ Hematopoietic and lymphoid cell neoplasm ▪ 3 more	2	2020
Defactinib (VS-6063)	NCT02465060	Targeted Therapy Directed by Genetic Testing in Treating Patients With Advanced Refractory Solid Tumors, Lymphomas, or Multiple Myeloma (The MATCH Screening Trial)	Active, not recruiting	▪ Advanced malignant solid neoplasm ▪ Bladder carcinoma ▪ Breast carcinoma ▪ 47 more	2	2015
Defactinib (VS-6063)	NCT02407509	Phase I Trial of VS-6766 Alone and in Combination With Everolimus	Recruiting	▪ Solid tumors ▪ Multiple myeloma ▪ Lung cancer ▪ 1 more	1	2015
VS-4718	NCT02215629	Dose Escalation Study in Acute Myeloid or B-Cell Acute Lymphoblastic Leukemia	Withdrawn	▪ Relapsed or refractory acute myeloid leukemia ▪ Relapsed or refractory acute lymphoblastic leukemia	1	2014

The search was performed in the ClinicalTrials.gov database putting “Defactinib” or “VS-4718” as OTHER TERM and “Leukemia” or “Lymphoma” or “Multiple Myeloma” or “Myelodysplastic Syndromes” as CONDITION/DISEASE.

**Figure 2:**
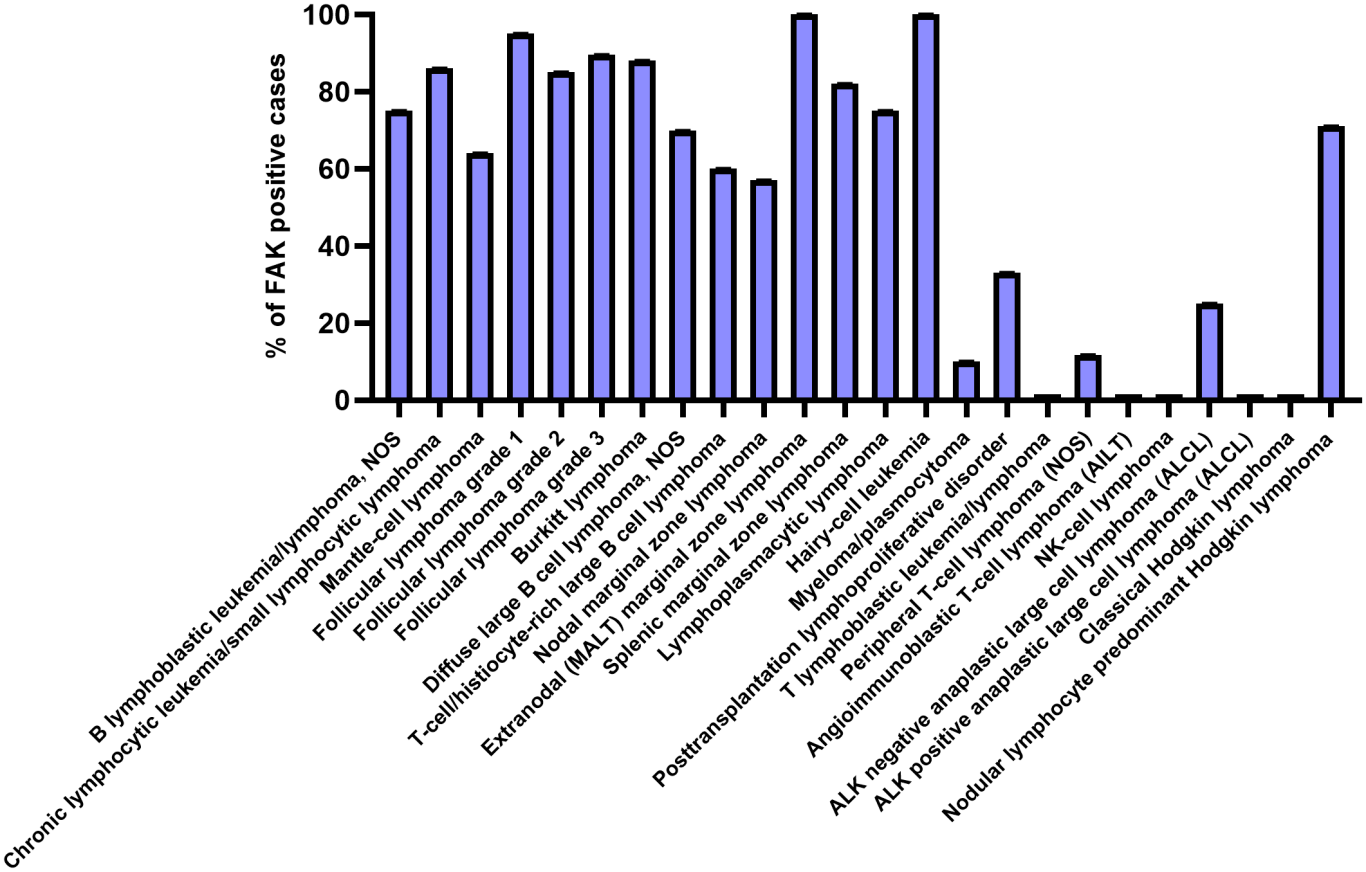
FAK expression in the main hematological diseases. Each bar represents the percentage of positive cases for FAK expression in the various blood/lymphoid disorders. The graph has been designed using GraphPadPrism software, starting from the immunohistochemistry data of (19).

In 2009, Ozkal and colleagues measured FAK protein expression levels, by immunohistochemistry, in the main human hematological diseases (**Figure 2**). In particular, FAK was expressed most highly in B-cell lymphomas, whereas T-cell lymphomas were predominantly negative for FAK expression. Regarding Hodgkin lymphomas, FAK was detected only in the tumor cells of the lymphocyte-predominant type (19).

In this review, we will discuss the main evidence related to FAK in the different hematological diseases, namely leukemia, lymphoma, multiple myeloma, and myelodysplastic syndromes.

## 2 FAK in leukemias

### 2.1 Chronic lymphocytic leukemia

Chronic lymphocytic leukemia (CLL) is the most common form of adult leukemia in western countries, with a median age at diagnosis of 72 years (20) and a global incidence of 100,000 new cases in 2019 (21). This lymphoproliferative disease occurs due to the accumulation of small mature B cells typically expressing the CD5, CD19, and CD23 surface antigens (22). Several prognostic markers have been established, with the mutational status of the immunoglobulin heavy chain variable region (IGHV) and the presence/absence of *TP53* gene deletions and/or mutations being the most reliable (23). CLL is a very heterogenous disease in which genetic lesions, together with other factors, determine the clinical outcome of the patients (20). In recent years, the survival of patients with CLL has increased due to the introduction of new targeted therapies (21); despite this, CLL is still an incurable disease.

As far as FAK in CLL is concerned, only a few studies are currently present in the scientific literature. Sbrana et al. recently showed that FAK mRNA is expressed less in CLL cells than in healthy B cells and is correlated with a progressive disease. They showed that *in vitro* treatment of primary CLL cells with defactinib caused a dose-dependent increase in cellular apoptosis (significant at a dose of 4 µM). Defactinib has been revealed to also be effective in an Eµ-TCL1 CLL mouse model, in which the *in vivo* treatment (75 mg/Kg, twice per day for 3 weeks) reduced the amount of leukemic cells circulating in the peripheral blood or resident in the bone marrow (BM), spleen, and intraperitoneum (24). In another *in vitro* study, through RNA-Seq analysis and using a dynamic circulating model, Burley et al. showed that more than 3,000 genes were altered when CLL cells underwent transendothelial migration, with an overrepresentation of genes involved in adhesion and migration; in particular, an upregulation of the FAK signaling pathway has been observed. These data were further confirmed by the detection through qRT-PCR of a higher expression of FAK in CLL migrating cells. Additionally, migration assays through Transwell, with CXCL12 chemokine as a chemo-attractive agent, showed how 5 µM of defactinib could reduce the migration/cell invasion of CLL cells; the combination of defactinib with the Bruton’s tyrosine kinase (BTK) inhibitor ibrutinib (both drugs were used at 2.5 µM) had a synergistic effect in decreasing the migration of CLL cells compared with the use of the single agents (25). It has recently been demonstrated that, in *in vitro* primary CLL cells, FAK is activated after B-Cell Receptor (BCR) stimulation. This event promotes calcium-mediated calpain activation that, in turn, induces cleavage and the phosphorylation/activation of FAK, associated with a downmodulation of the full-length FAK form. In particular, FAK activation was higher in patients with an unmutated status of immunoglobulin heavy chain variable region genes (UM-IGHV), which is related to poor prognosis (26). In the same group of patients, the CLL-related proteins cortactin and HS1 (hematopoietic lineage cell-specific protein 1) (27, 28), which can interact with FAK and are involved in cytoskeletal shaping and motility of the tumoral cells, were overexpressed, thus hypothesizing a possible interplay with FAK in CLL. FAK activation upon BCR engagement had been already demonstrated on primary CLL cells by Lòpez-Guerra et al., who also showed how this process can be inhibited by the multi-kinase inhibitor sorafenib (10 µM) (29). In conclusion, FAK can be considered an important player in CLL pathogenesis and, therefore, it could become an innovative therapeutic target in the development of new pharmacological treatments of this disease. Further research focusing on the full-length and cleaved forms of FAK could be important in better understanding the activation and function of this protein.

### 2.2 Acute myeloid leukemia

Acute myeloid leukemia (AML) is the most common form of acute leukemia in adults and is usually diagnosed at approximately 70 years of age (30). In western countries, the annual AML incidence is 30/40 cases per million people (30). AML incidence and mortality were both increased between 1990 and 2019, with a mortality-to-incidence ratio showing a decrease in the same period, thus indicating that the survival chances for AML patients have progressively increased over the years (31), especially correlating with the introduction of novel therapies beyond the chemotherapeutic regimen (32). AML originates from alterations occurring in immature myeloid precursors, namely myeloblasts, which present impaired differentiation potential, proliferate, and abnormally accumulate in the bone marrow and the peripheral blood (30). Typical AML cell markers are MPO, CD13, CD33, CD65, and CD117, whereas examples of molecular markers are t(8;21) and t(6;9) aberrations indicating a favorable and an adverse prognosis, respectively (30).

**Figure 3:**
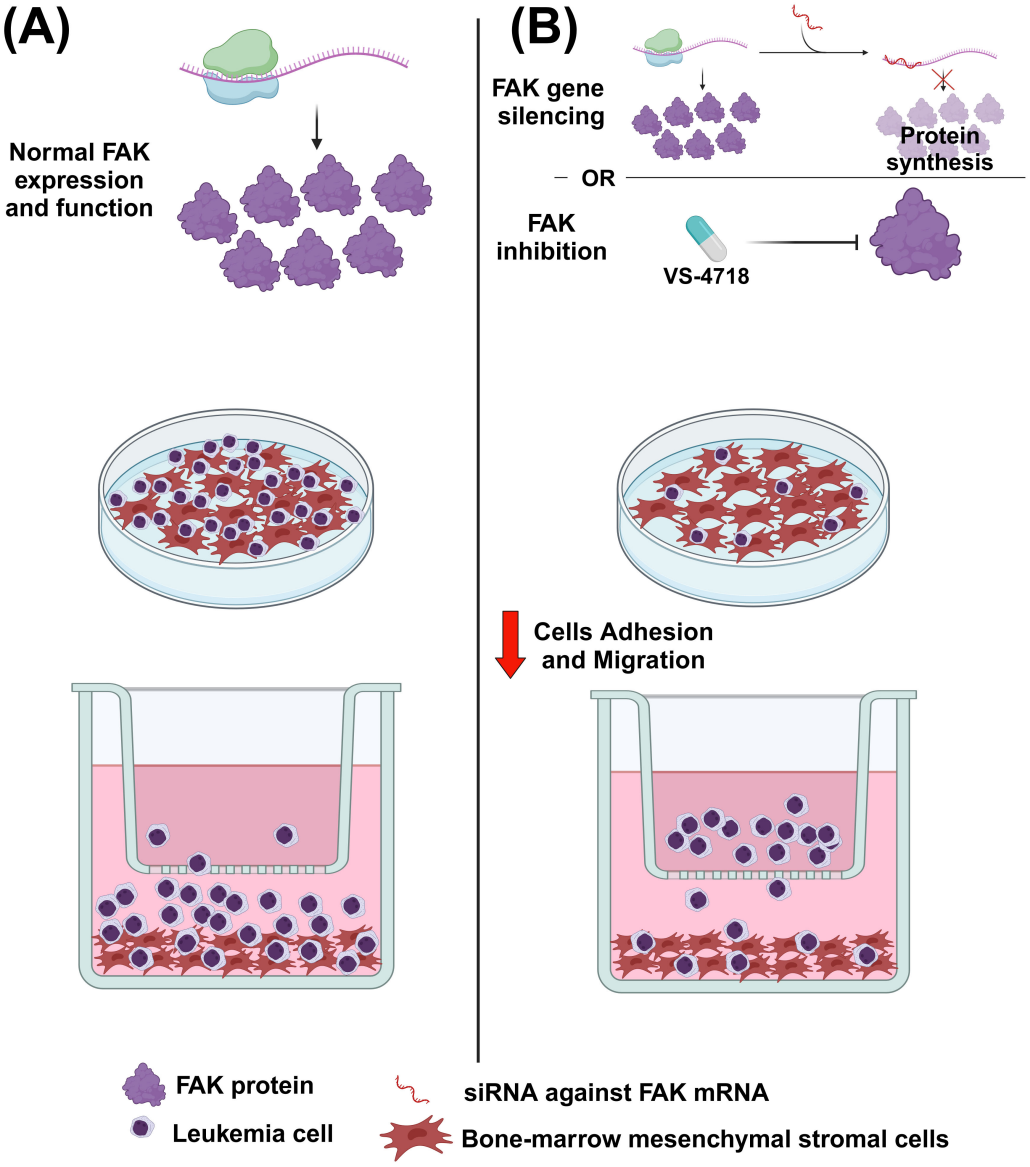
The effect of FAK inhibition or FAK gene silencing on acute myeloid leukemia (AML) cells. **(A)** In normal conditions, AML cells can migrate and adhere to mesenchymal stromal cells (MSCs). **(B)** After administering the FAK inhibitor VS-4718 or using RNA interference against FAK, the migration and adhesion of AML cells to stromal cells is reduced. Image created with Biorender.com (under the license of the University of Padua).

FAK expression (analyzed *ex vivo* by flow cytometry) has been revealed to be, together with the expression of CXCR4 (CXC chemokine receptor type 4) and integrin VLA-4 (very late antigen-4), an important negative prognosticator of the clinical outcome of AML (33). This result was supported by another study in which a high FAK expression in the peripheral blood and bone marrow of AML patients was associated with unfavorable cytogenetics (34). A mechanism to explain the higher FAK expression in high-risk AML patients has been proposed. FAK gene expression can, in fact, be negatively regulated by two miRNAs (i.e., hsa-let7a-2-3p and hsa-miR-135a-5p), which are in turn regulated by RUNX-1 (runt-related transcription factor 1), which is often mutated in the high-risk AML subset. Therefore, the idea is that RUNX1 upregulates the two miRNAs that, in turn, decrease FAK expression, thus making AML cells less sensitive to FAK inhibitors. Consistently, the authors showed that a decreased expression of hsa-let7a-2-3p and hsa-miR-135a-5p made AML cells more sensitive to FAK inhibitors VS-4718 (1.5 µM) and defactinib (1 µM) (35). Owing to alternative splicing, FAK can be present in different isoforms that are involved in AML prognosis. The canonical form is FAK^0^, but other forms also exist, namely FAK^28^, FAK^6^, and FAK^7^ (overall assumed as FAK^6*^). In AML, it has been demonstrated that, compared with FAK negative cases (FAK^0^), the FAK^6*^ patients are associated with a shorter overall survival, event-free survival, and length of clinical remission. Therefore, in AML, the expression of FAK variants is associated with a poor prognosis. Mechanistically, it has been demonstrated that the FAK^6*^ isoforms are associated with β-catenin and Wnt5a signaling, thus leading to self-renewal, proliferation, and the survival of leukemic stem cells (36). In another study, researchers showed evidence of the importance of FAK in AML biology. First, the treatment with FAK inhibitor VS-4718 (0.2–1 µM) of an AML cell line reduced FAK expression, as detected by western blotting analysis. The same treatment also decreased the expression of the anti-apoptotic proteins MCL-1 (induced myeloid leukemia cell differentiation protein 1) and BCL-XL (B-cell lymphoma extra-large). Furthermore, VS-4718 (0.1–1 µM) combined with the BCL-2 (B-cell lymphoma 2 protein) inhibitor venetoclax (13–200 nM) had a synergistic effect in inducing the apoptosis of AML cell lines, even in co-culture conditions with mesenchymal stromal cells (MSCs), which mimic the pro-survival support from the tumor microenvironment (37–39). Similarly, primary AML cells from patients’ bone marrow showed increased apoptosis when exposed to the drug combination VS-4718 (0.2-2.5 µM) + venetoclax (1.3–20 nM) with respect to the single agents. Finally, the authors showed how FAK gene silencing made AML cell lines more sensitive to the treatment with venetoclax (12.5–200 nM). In the same study, in an AML mouse model, they demonstrated how VS-4718 (75 mg/kg, twice per day) and venetoclax (100 mg/kg, daily), alone or in combination, owned anti-leukemic effects in terms of decreasing circulating AML cells and longer survival with respect to untreated mice (37). FAK can also have an impact in the tumor microenvironment of AML; indeed, the previously cited VS-4718 FAK inhibitor (1.25–2.5 µM) and FAK gene silencing were able to induce a decrease in the adhesion and migration of OCI-AML3 cells to bone-marrow derived MSCs, thus suggesting how FAK inhibition can affect leukemia cells-stroma interaction (**Figure 3**). Moreover, *in vivo* administration of VS-4718 (75 mg/kg, twice per day for 16 days) in an AML mouse model reduced the leukemia burden, decreased the number of CD45+ cells in peripheral blood, and reduced the tissue infiltration of leukemia cells (34).

### 2.3 Acute lymphoblastic leukemia

Acute Lymphoblastic Leukemia (ALL) is a blood disease that affects children more frequently than adults, with a peak of incidence between 1 and 4 years of age (40). Regarding ALL incidence, this parameter has more than doubled in the last 30 years, going from 66,810 cases in 1990 to 153,320 cases in 2019 globally. On the contrary, in the same period, overall deaths were relatively stable with a decreased trend, indicating, as for the other diseases, an improvement in ALL treatment and management (41). As far as ALL pathogenesis is concerned, leukemic cells derive from an abnormal proliferation of immature lymphoid precursor stem cells, which can spread mainly in the bone marrow and blood but also in extramedullary sites (40). Considering the lymphoid origin of these malignant cells, immunophenotyping is essential for discriminating between ALL from T-cell (T-ALL; CD3, CD5, CD7, and TdT) (42) or B-cell lineages (B-ALL; CD19, CD22, CD10, and CD34) (43). Trisomy of chromosomes 4, 10, or 17 and *MLL* gene rearrangements are favorable and adverse markers, respectively (40).

In T-ALL, the VLA-4 integrin was shown to have a role in chemoresistance. In particular, the binding of VLA-4 to its ligand VCAM-1 (vascular cell adhesion molecule 1) was able to interrupt doxorubicin-mediated cell death in T-ALL cell lines. The VLA-4/VCAM-1 binding induced the phosphorylation/activation of the FAK homologous protein PYK2 (proline-rich tyrosine kinase 2) and a consequent drug efflux; moreover, inhibiting PYK2 (VS-6063, 0.5 µM) the signaling cascade downstream VLA-4 activation was stopped with the consequent inhibition of doxorubicin efflux and chemoresistance (44). A preclinical *in vivo* study by You et al. showed a link between FAK and the phosphatase PTEN (phosphatase and tensin homolog) in T-ALL. As a background, PTEN protein is downmodulated in 15–25% of T-ALL patients due to genetic mutations, resulting in an upregulation of the PI3K-AKT-mTOR pathway (phosphatidylinositol 3-kinase/protein kinase B/mechanistic target of rapamycin), which provides a survival stimulus for leukemic cells and, for this reason, PI3K inhibitors have started to be used in the clinical management of T-ALL. Nevertheless, some patients reach only partial remission under PI3K inhibitors-containing regimens. You and colleagues discovered that this was due, at least in part, to the parallel activation of the FAK-NF-kB pathway (nuclear factor kappa-light-chain-enhancer of activated B cells), which leads to the expression of pro-survival molecules in leukemic cells, such as BCL-2 and BCL-XL. Further experiments in mice demonstrated that the genetic or pharmacological (PF, a FAK inhibitor, at 50 mg/kg for 26 days) targeting of FAK in PTEN null T-ALL cells made the latter more sensitive to the treatment with PI3K inhibitors (45).

As far as B-ALL is concerned, qRT-PCR and western blotting analyses revealed that FAK is more highly expressed in primary leukemic cells than in healthy lymphocytes. The authors showed how in the REH B-ALL cell line, FAK gene silencing could increase the effectiveness of the mTOR inhibitor rapamycin in suppressing cell proliferation, cell cycle arrest, and apoptosis. Additionally, the combination of rapamycin with FAK downmodulation in REH cells, after being injected into mice, produced a positive effect in terms of survival and leukemia arrest (46). In an *in vivo* study, FAK was upregulated in murine Philadelphia chromosome positive (Ph+) B-ALL cells. Moreover, the FAK inhibitor VS-4718 (at a concentration of >0.1 µM) was cytotoxic in murine and human Ph+ B-ALL cells. In addition, VS-4718 (50 mg/kg) had a synergistic effect in combination with dasatinib, (a tyrosine kinase inhibitor targeting Src and BCR/ABL - breakpoint cluster region-Abelson murine leukemia kinase - widely used in frontline treatment of Ph+ B-ALL) in affecting cell survival, adhesion, and inhibition of downstream targets of FAK (47). TAE226, another FAK inhibitor, was revealed to be useful in treating Ph+ B-ALL. In particular, in a Ph+ B-ALL cell line, it has been demonstrated that this drug affected cell proliferation in a dose-dependent manner (IC50 = 0.26 µM). However, TAE226 (30 mg/kg) as single agent did not significantly compromise the *in vivo* tumor growth in mice; nevertheless, in the same mice, TAE226 was effective if it was combined with the BCR-/ABL tyrosine kinase inhibitor nilotinib (48). Considering this evidence, the combined inhibition of BCR/ABL and FAK kinases could represent a new therapeutic option for Ph+ B-ALL patients. FAK was also important in the response to imatinib, another BCR/ABL kinase inhibitor used for the treatment of Ph+ B-ALL. Indeed, Le et al. showed that murine BCR/ABL BaF3 cells with silenced FAK had a reduced growth and proliferation rate and a higher tendency to undergo apoptosis. Moreover, mice injected with these cells had longer survival and less leukemia progression than mice injected with BCR/ABL BaF3 cells with wild-type FAK. Coming back to the BCR/ABL inhibitor imatinib, BCR/ABL BaF3 cells with silenced FAK were more sensitive to the treatment with this drug both *in vitro* (5 and 50 µM) and *in vivo* (in mice, 25 mg/kg) (49).

## 3 FAK in lymphomas

### 3.1 Mantle cell lymphoma

Non-Hodgkin lymphomas (NHLs) represent approximately 90% of all lymphomas and have a plethora of different pathological condition subtypes (50). Among them, 3–10% are represented by mantle cell lymphoma (MCL), usually diagnosed between the ages of 60 and 70 and recruiting approximately 4–8 new cases per million people per year (data referred to the USA) (51). MCL cells could originate from the neoplastic transformation of naïve pre-germinal center B cells or from antigen-experienced post-germinal center/memory B cells, inducing an aggressive or indolent disease, respectively (51). In recent years, the introduction of new targeted treatments has significantly improved the overall survival of MCL patients (52). MCL has a distinct immunophenotype, expressing CD5, BCL-2, CD20, IgM/IgD, CD22, and CD79, being negative for CD10 and BCL-6, and overexpressing cyclin D1 [due to t(11; 14) (q13; q32) translocation, a genetic hallmark of MCL] and the transcription factor SOX11 (53–55). Moreover, insights into the clinical outcome of MCL patients come from some biological/molecular markers, such as a high proliferation rate (Ki67 > 30%) and a high p53 expression, with these features typically associated with a shorter overall survival (55).

As previously shown in **Figure 2**, FAK is highly expressed in MCL. Indeed, these data have been confirmed by Rudelius et al. by immunohistochemistry of bone marrow infiltrates obtained from MCL patients and by western blotting analysis of MCL cell lines; in the latter, pFAKY397 was also found to be highly expressed. These authors also showed how the chemokine CXCL12 could induce FAK and pFAKY397 upregulation in MCL cell lines; in particular, as CXCL12 is highly expressed in MSCs from MCL bone marrow (BM-MSCs), the co-culture between stromal cells and MCL cell lines, or primary MCL cells, identified an upregulation of the FAK signaling pathway, such as AKT, ERK1/2, and NF-kB. Subsequently, Rudelius and colleagues used a loss-of-function approach to study the role of FAK in MCL cells. They showed that FAK inhibition with VS-6063 (100 nM) or FAK gene silencing led to an inhibition of FAK downstream targets and *in vitro* invasiveness/migration capacity toward CXCL12. Moreover, the treatment of some MCL cell lines resistant to the Btk inhibitor ibrutinib in co-culture with MSCs with escalating doses of Ibrutinib and VS-6063 (0-1 µM) demonstrated a synergistic anti-tumor effect (combination index < 0.6) (56). This study demonstrated that the FAK inhibitor VS-6063 can overcome ibrutinib resistance in MCL. In another study, the authors demonstrated, through chromatin immunoprecipitation (ChIP) and luciferase assays, how the SRY-related HGM-box transcription factor 11 (SOX11) could bind the *PTK2* gene and upregulate FAK expression in MCL cell lines, thus inducing the activation of the AKT and ERK1/2 pathways. In line with these results, SOX11^pos^ MCL cells have been shown to increase cell migration, transmigration through endothelial cells, adhesion to stromal cells, cell proliferation, and resistance to conventional pharmaceutical treatments compared with SOX11^neg^ MCL cells. By contrast, FAK inhibition with PF-573228 (10 µM) decreased the intensity of all these aspects to levels comparable with those shown by SOX11^neg^ MCL cells. In a mouse model, the same researchers demonstrated how SOX11 expression promoted MCL cell migration and, consequently, the organ dissemination of tumor cells, with all events decreasing following treatment with the FAK inhibitor PF-573228 (30 mg/kg for 28 days) (57). Overall, SOX11 has been proven to play an important role, together with FAK, in determining an aggressive phenotype in MCL cells. Another interesting point was the link found by Zhang et al. between Hedgehog (Hh) and FAK in MCL (58). Hh signaling was demonstrated to favor the growth and dissemination of tumor cells in solid (59–63) and hematological cancers (64–66). In this context, Zhang and colleagues tested the *in vitro* effects of the Hh inhibitor LDE225 on peripheral blood mononuclear cells (PBMCs) from MCL patients and in MCL cell lines. They showed that this drug (10–30 µM) could decrease the expression of FAK and integrin VLA-4 in MCL cells. This finding also explained why LDE225 treatment decreased MCL cell adhesion to BM-MSCs and migration *in vitro* (58).

### 3.2 Diffuse large B-cell lymphoma

Among the various forms of NHL, the most common is diffuse large B-cell lymphoma (DLBCL) (50), representing between 25 and 40% of NHLs (67). As for DLBCL incidence, 7.2 new cases each 100,000 people have been registered in the USA (68). A recent Brazilian study demonstrated that the 5-year overall survival in DLBCL is approximately 50% (69); coherently, approximately 60% of patients can reach complete disease remission after a standardized immunochemotherapy regimen (70). This disease originates from the tumoral transformation of B cells, with DLBCL represented by two main subtypes: i) the germinal B-cell like (GCB), expressing CD10 and BCL2 rearrangements, and ii) the activated B-cell like (ABC), the most aggressive form that is derived from post-germinal center B cells with B-cell Receptor dependence, constitutive NF-kB activation, and IRF4/MUM1 expression (71). DLBCL can be diagnosed by cell positivity for CD19, CD20, CD22, CD45, CD79a, and IgM by immunohistochemistry or flow cytometry (72). Increased p53 expression or the double expression of MYC transcription factor and anti-apoptotic BCL-2 protein are predictors of a poor prognosis, and CD30 positivity accounts for a better clinical outcome (67).

In DLBCL, FAK is highly expressed (**Figure 2**) and has been revealed to be a valid positive prognostic factor, considering that higher FAK expression (by immunohistochemistry) is associated with longer overall survival and progression-free survival with respect to the cases with a lower FAK expression; therefore, FAK could be a new molecular marker that is useful for refining the risk stratification of DLBCL patients (73). The effects of the compound E7123, a celecoxib derivative, were tested in DLBCL. It was shown that E7123 had a negative impact on DLBCL cell line viability (IC50 range, 13.08–17.66 µM). Similarly, in a mouse model, E7123 suppressed tumoral growth (100 mg/kg, for 22 days). In addition, it has been demonstrated that E7123 (60 µM) can reduce FAK expression in DLBCL cell lines. However, the genetic overexpression of FAK in DLBCL cell lines made these cells more resistant to E7123 treatment, as demonstrated by the higher viability of these cells compared with wild-type DLBCL cell lines (74). Therefore, FAK expression was also shown as a key determinant for the response to E7123 treatment in DLBCL.

### 3.3 Non-Hodgkin lymphoma in general

**Figure 4:**
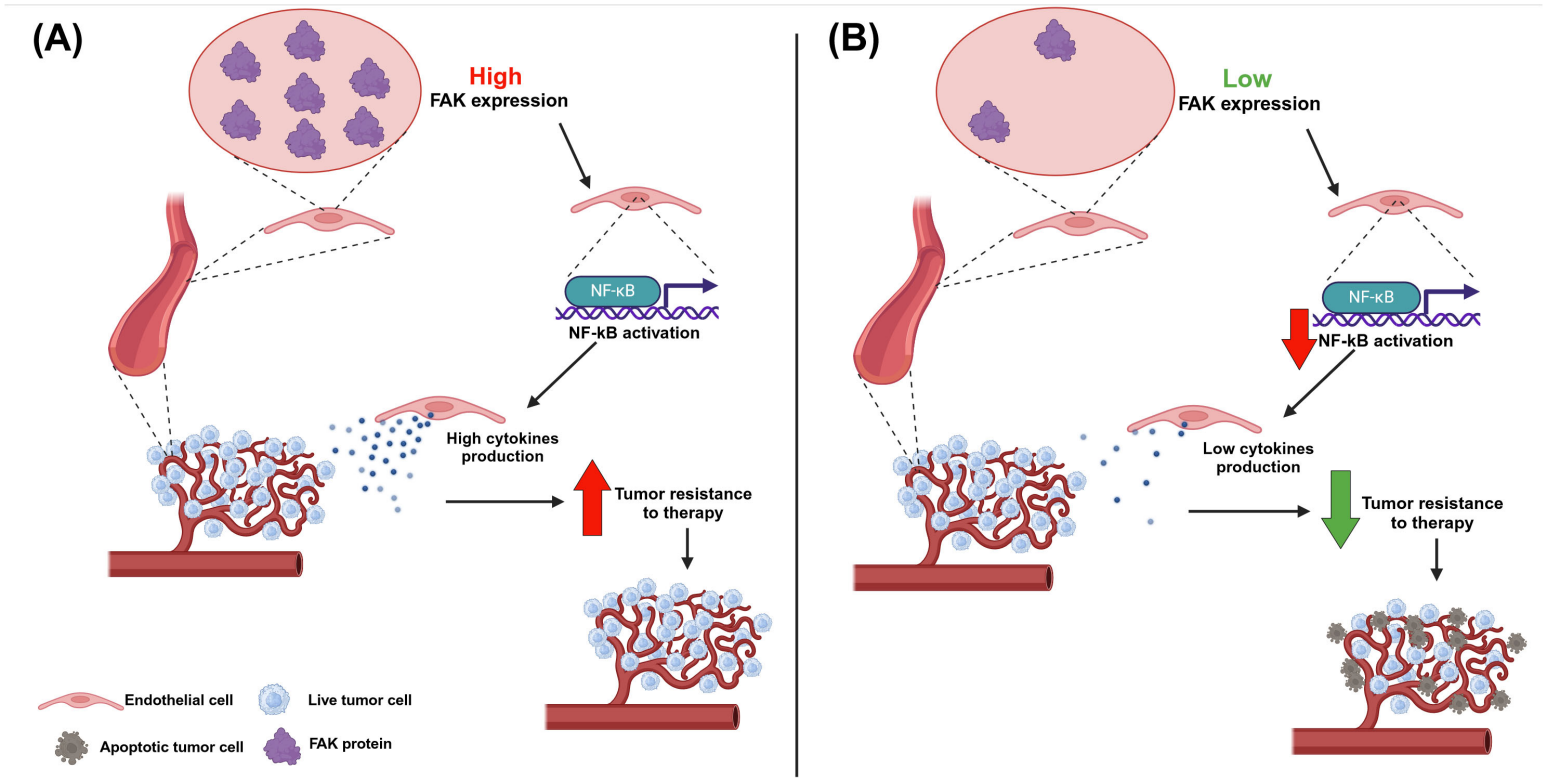
The effect of FAK expression in blood vessels in non-Hodgkin lymphoma. **(A)** If endothelial cells express FAK this, in turn, leads to the activation of the NF-kB transcription factor, which promotes the production of cytokines; these last ones provide support to the tumor cells, which become more resistant to DNA-damaging therapies. Therefore, high FAK expression in blood vessels is correlated with a poor prognosis in non-Hodgkin lymphoma. **(B)** If endothelial cells do not express or express low levels of FAK this, in turn, leads to a decrease in the activation of the NF-kB transcription factor, with a consequent reduction in cytokine production; in this case, tumor cells miss the support from the blood vessel cells and are more sensitive to DNA-damaging therapies. Therefore, low or null FAK expression in blood vessels is correlated with a good prognosis in non-Hodgkin lymphoma. Image created with Biorender.com (under the license of the University of Padua).

In the context of the tumor surrounding microenvironment, FAK has been revealed to be important in the endothelial cells of patients affected by NHLs, which are generally considered to be solid tumors derived by different immune system cell types (75). In particular, after doxorubicin administration, cases with a low FAK expression (assessed through immunohistochemistry in human lymphoma sections) in endothelial cells underwent a complete remission of disease. Conversely, cases with higher FAK expression in blood vessels were associated with disease progression. In line with this evidence, other experiments showed how FAK deletion in endothelial cells determined an augmented cell death and a decreased cell proliferation of tumor cells within perivascular compartments in mice treated with doxorubicin and radiotherapy. Further *in vitro* and *in vivo* analyses enabled the creation of a model to explain this increased sensitization to DNA-damaging therapies in cases with FAK-null endothelial cells; specifically, FAK could activate the transcription factor NF-kB in endothelial cells, thus inducing the production of cytokines (i.e., C5, GM-CSF, IL-1α, IL-2, IL-4, IL-6, IL-16, KC, MIG, MIP-2, and TIMP-1), which can support the tumor. In the absence of FAK within blood vessels, this mechanism fails and the tumor becomes more sensitive to DNA damaging-therapies, such as doxorubicin treatments (**Figure 4**). Therefore, efforts to develop new therapies targeting FAK in endothelial cells should be undertaken (76).

## 4 FAK in multiple myeloma

Multiple myeloma (MM) represents approximately 10% of all hematological diseases (77). The age range of MM diagnosis is between 65 and 75 years. A total of 155,688 new cases were registered globally in 2019, with an incidence rate of 1.92 new cases per 100,000 people. In 2020, 117,077 worldwide deaths from MM were reported (a mortality rate of 1.14 deaths per 100,000 people). However, an overall increase in the 5-year survival rate from 25% in 1975–1977 up to 60% in 2012–2018 in the USA and Europe was described, mainly thanks to the novel therapies entry the MM management (78). MM derives from the malignant transformation of plasma cells (differentiated B cells that can produce and release soluble antibodies) mainly localized in the bone marrow (79) but which can also move in the peripheral blood and other extramedullary sites (78). Flow cytometry detection of markers such as CD19, CD38, and CD81 can help in defining the MM cell population (80), and disease outcome is predicted by several biomarkers such as the deletion of chromosome 17p, which is associated with a poor prognosis (81).

Focal adhesion proteins, such as FAK and PYK2, are also important in the context of MM. It has been demonstrated that FAK mRNA expression levels were significantly higher in MM patients with stage III with respect to patients with stage I+II; moreover, patients with extramedullary infiltration, a condition referred to as neoplastic plasma cells penetration within bones and other organs, express a significantly higher level of FAK mRNA with respect to MM patients without infiltration. In addition, a similar difference has been demonstrated at the protein level. Overall, FAK might be responsible, at least in part, for disease progression and extramedullary infiltration in MM (82). An interesting pharmacological study showed how asiatic acid (AA, a pentacyclic triterpene, used at 40 µM) can interrupt cell cycle progression in the RPMI 8226 MM cell line, decreasing the expression level of FAK and pFAK (hence the activated form), thus highlighting a possible mechanism of action of AA in MM involving FAK (83). Regarding the FAK homolog PYK2, it has been shown that AKT, JAK1 (janus kinase 1), and PYK2 phosphorylation increased in MM cell lines after adhesion to bone marrow-derived MSCs. Specifically, this interaction with tumor stromal partners favors cell survival, whereas inhibiting or downmodulating PYK2 with RNA interference resulted in increased apoptosis in MM cells co-cultured with MSCs (84). Another study demonstrated how PYK2 is important for MM cell biology. Zhang et al. showed that primary MM cells expressed higher PYK2 mRNA levels than plasma cells from healthy individuals (*vice versa* for FAK—data obtained from the analysis of the published gene expression dataset GSE2658). These data have also been confirmed at the protein level with immunohistochemistry on bone marrow samples from MM patients and healthy donors. PYK2 gene silencing determined a reduction in MM cell proliferation, adhesion, and cell-cycle progression by suppressing Wnt/β-catenin signaling; additionally, RNA interference targeting PYK2 decreased MM tumor growth and stretched the survival time in a xenograft mouse model. Conversely, overexpression of PYK2 in MM cells favored tumor progression and decreased survival in the same model. Finally, the authors tested the effects of the FAK/PYK2 inhibitor VS-4718 (10 µM) in MM cells; this compound inhibited MM cell proliferation *in vitro* to exert cytotoxic activity and inhibit cell migration. The same drug also reduced tumor growth in mice (75 mg/kg for 28 days) (85). Other authors focused on the study of hypoxia in MM cells (86). Hypoxia is a condition of oxygen deprivation commonly found in the inner part of many tumors. This condition has been demonstrated to induce resistance to proteasome inhibitors (e.g., Bortezomib) in MM cells (87). Researchers showed that this hypoxia-mediated drug resistance could be reversed by treating MM cells with the PYK2/FAK inhibitors VS-4718 and VS-6063, thus increasing the rate of cell death. Moreover, using the Chou–Talalay method to calculate combination indexes, the authors demonstrated an additive effect in decreasing MM cell survival for the combination of bortezomib (5 nM) with VS-4718 or VS-6063 (2.5 µM). In line with these results, survival appeared to be longer in an MM mouse model treated with bortezomib (0.5 mg/kg, biweekly) plus VS-4718 or VS-6063 (50 mg/kg, twice daily) with respect to the treatment with bortezomib as a single agent (86). Overall, FAK inhibitors can represent a new therapeutic chance for all MM patients resistant to bortezomib treatment.

## 5 FAK in myelodysplastic syndromes

Myelodysplastic syndromes (MDS) consist of a various repertoire of clonal hemopoietic stem cell disorders in which an alteration of physiological hematopoiesis, due to the accumulation of genetic mutations, determines peripheral cytopenia and the augmented risk of leukemia transformation (88). In this context, the bone marrow microenvironment plays a central role in the development of MDS (89–91); in particular, genetic or functional alterations of MSCs are implied in the occurrence of MDS (90, 92–94). MDS represents relative common malignant diseases with an overall incidence of approximately 4 cases per 100,000 people per year and is age-related considering that MDS typically appears around the age of 70 years (95). The 50% of MDS patients can survive up to 3 years after initial diagnosis, also considering that there is a general 30% risk that MDS transforms in AML (95). Differently from other blood cancers, in MDS bone marrow aspirates and/or biopsy result critical for MDS diagnosis and classification, whereas immunophenotyping analysis has a diagnostic value still debated in scientific community (88). However, there is evidence that immunophenotypic markers (such as CD7, CD11b, CD22, CD33, CD44, CD45RA, CD56, CD123, CD366 or CD371) aberrantly expressed on hematopoietic stem cells in MDS are associated with a higher risk of leukemia advent (96). Considering prognosis, several genetic abnormalities can stratify MDS patients, such as deletion of chromosome 7p or 12p, associated to unfavorable and favorable clinical outcome, respectively (88).

**Figure 5:**
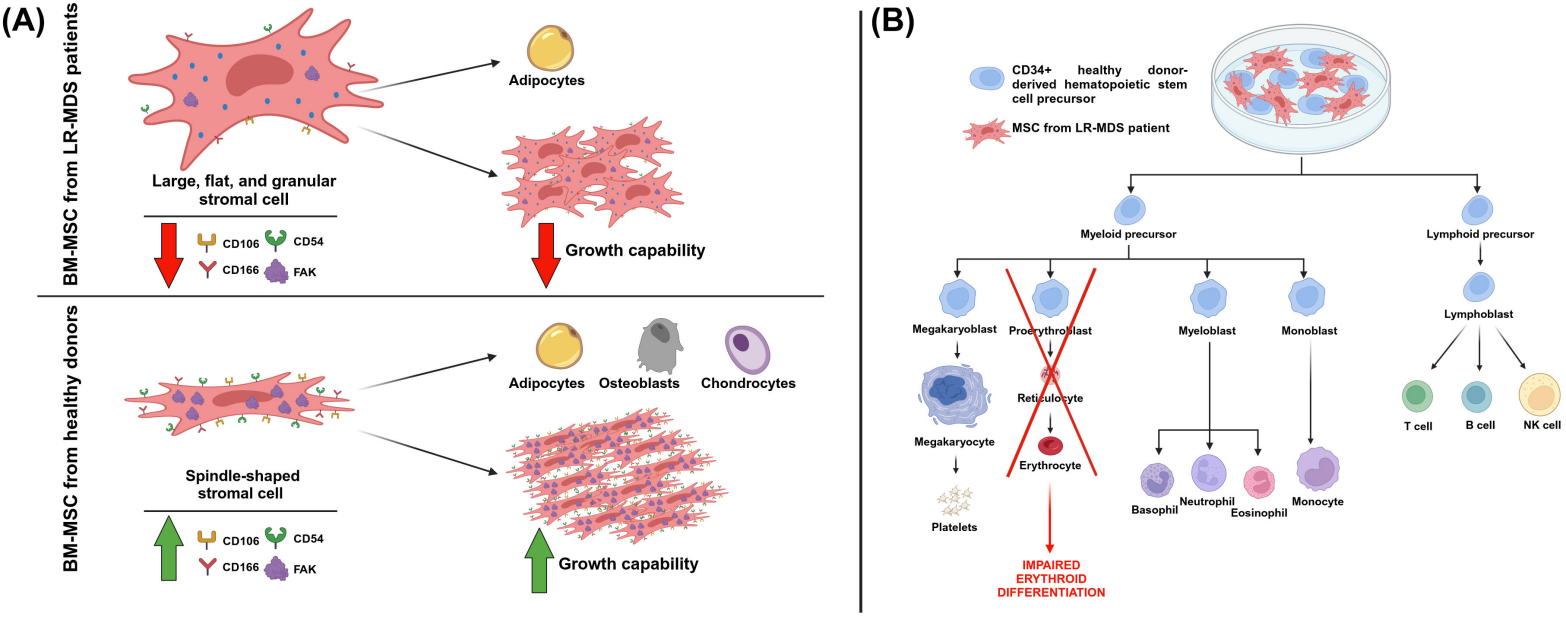
The effects of FAK deficiency in mesenchymal stromal cells (MSCs). **(A)** MSCs from healthy donors and low-risk myelodysplastic patients (LR-MDS) show different morphologies and functionality. Regarding this last aspect, in MSCs from LR-MDS patients, there is a lower expression of FAK and of the markers CD106, CD166, and CD54. Moreover, these cells are less prone to proliferation, whereas they prefer the adipogenic differentiation lineage. **(B)** The effect of the co-culture of MSCs from LR-MDS patients with CD34+ healthy donor (HD)-derived hematopoietic stem precursor cells (HSPCs), resulting in an impairment of erythroid differentiation. Image created with Biorender.com (under the license of the University of Padua).

In the context of MDS, FAK has been found to be downregulated, in terms of both expression and activation, in MSCs of low-risk MDS patients (LR-MDS). Moreover, in comparison with MSCs from healthy donors, MSCs from LR-MDS patients had a lower growth capability and a differentiation program more prone toward adipogenesis (**Figure 5A**) (97). In a further study, Wu et al. characterized the effects of FAK deficiency in bone marrow MSCs (BM-MSCs) on hematopoiesis. The decreased FAK expression in BM-MSCs from LR-MDS patients was correlated with morphological and functional changes. Regarding shape mutations, BM-MSCs had large, flat, and granular stromal cells compared with the spindle-shaped MSCs cells from healthy donors derived from bone marrow aspirates (**Figure 5A**). As far as functional changes are concerned, BM-MSCs showed a decreased expression of CD106, CD166, and CD54 (**Figure 5A**). Moreover, FAK deficiency in BM-MSCs from LR-MDS patients has been associated with a decreased level of hemoglobin, with a positive correlation found between the *PTK2* gene expression level and blood hemoglobin level. The same authors created an *in vitro* model of FAK deficiency in MSCs by downregulating FAK with RNA interference in an HS-5 stromal cell line. In this model, they noticed altered morphology, proliferation, differentiation capabilities, and the expression of several adhesion molecules. Eventually, authors showed that, when co-cultured with FAK-deficient BM-MSCs, the CD34+ healthy donor (HD)-derived hematopoietic stem precursor cells (HSPCs) were characterized by abnormal proliferation and impaired erythroid differentiation (**Figure 5B**) (98). In light of all this evidence, we can conclude how a defective microenvironment, in particular a FAK-deficient stroma, is directly involved in the impaired hematopoiesis that characterizes MDS.

## 6 Discussion and conclusion

In this review, we explored the intricate role of FAK in various onco-hematological diseases, shedding light on its diverse functions in different contexts.

In the specific onco-hematological field, FAK involvement has been highlighted in several types of leukemias and lymphomas. In CLL, the intricate interplay of FAK with BCR stimulation and its association with poor prognostic factors (UM-IGHV status) underline its significance in CLL pathogenesis (26, 29). Moreover, pharmacological studies with defactinib indicate FAK as a potential new therapeutic target in CLL (24–26). FAK has been revealed to also be important in AML. Here, high FAK expression is associated with a poor clinical outcome (33, 34); moreover, the expression of precise FAK splicing isoforms correlate with a poor prognosis (36). This pushed scientists to further investigate the function of FAK in AML cell behavior, and drug studies showed how FAK inhibition could be a valid therapeutic option for the treatment of AML; particularly, the combination between VS-4718 and the BCL-2 inhibitor venetoclax was revealed to have a synergistic effect (34, 37). In T-ALL, it has been shown that the VLA-4/PYK2 (the FAK homologous) axis causes doxorubicin resistance in leukemic cells and that PYK2 inhibition with defactinib could overcome this chemoresistance (44). Moreover, in PTEN negative T-ALL patients, FAK confers resistance to PI3K inhibitors through a NF-kB-dependent mechanism; nevertheless, promising *in vivo* studies showed how the pharmacological targeting of FAK can make the PI3K inhibitors more effective (45). As far as B-ALL is concerned, a FAK downmodulation has been shown to increase the efficacy of mTOR inhibitor rapamycin on cell proliferation and cell cycle suppression, as well as apoptosis (46). Additionally, FAK has been found to be upregulated in the Ph+ form of B-ALL (47). In that subset, FAK has been demonstrated to be implicated in different pharmacological treatments: i) VS-4718 was cytotoxic, especially if combined with the BCR/ABL kinase inhibitor dasatinib (47); ii) TAE226 was effective in combination with the BCR/ABL kinase inhibitor nilotinib (48); and iii) the BCR/ABL kinase inhibitor imatinib was more effective in B-ALL cells with downmodulated FAK (49).

The present review has also delved into FAK involvement in lymphomas. In MCL, FAK has been proven to be an important molecule involved in the aggressive phenotype, which is conferred by SOX11 positivity (57). Moreover, drug studies showed how defactinib can decrease the migration/invasiveness of MCL cells but, above all, the most interesting data is that defactinib could represent an alternative therapeutic option for ibrutinib-resistant MCL patients (56). Considering DLBCL, high FAK expression has been highlighted as a positive prognostic marker (73), and the drug E7123 has been proven to have an anti-tumor effect in DLBCL cells by decreasing FAK expression (74); however, these two results seem to contrast each other and, therefore, further research is required. Bosch and colleagues demonstrated that, beyond FAK, E7123 could deregulate the expression of other focal adhesion proteins, such as PYK2, p130Cas, HEF1, and Lyn. Hence, the overall anti-tumor effect of E7123 observed in DLBCL cells could be due to a broad action on different proteins within the lymphoma cell.

In MM, FAK association with disease progression and extramedullary infiltration has been elucidated (82). Also, the FAK homolog PYK2 has been demonstrated to be important for MM cells; gene silencing of PYK2 affected MM cell proliferation, adhesion, and cell cycle arrest and, *in vivo*, induced a decrease in tumor growth, prolonging mice survival (85). Regarding pharmacological studies on FAK in MM, the inhibitor VS-4718 has been proven to be effective against MM cells (85); in particular, it was revealed to be a good option for bortezomib-resistant MM patients (86).

This manuscript highlighted some evidence related to the role of FAK in the tumor microenvironment (TME) of hematological diseases. For instance, it has been shown how FAK induces the adhesion and migration of AML cells toward stromal cells (**Figure 3**), thus promoting an leukemia-stroma interaction that can confer protection from drugs and pro-survival stimuli to AML cells in the context of the bone marrow niche (34). Furthermore, endothelial cells were revealed to be an important element of the TME, especially in NHLs (**Figure 4**). It has been underlined how endothelial cells can support lymphoma cells releasing cytokines through a mechanism based on the functioning of the FAK/NF-kB axis (76). Eventually, FAK was also important in the pathogenesis of MDS, in which the constitutive FAK downmodulation present in MDS MSC (typical of low risk patients) has been associated with morphological and functional changes of these cells, thus affecting the normal hematopoiesis (**Figure 5**) and emphasizing the contribution of FAK to the development of MDS itself (97, 98). A summary of findings related to the role of FAK in the various onco-hematological diseases is provided in **Table 2**.

**Table 2:** Summary of the discoveries about the role of FAK in different onco-hematological diseases.

Blood disease	Related evidence about FAK	References
**Chronic lymphocytic leukemia (CLL)**	FAK inhibition revealed to be effective against CLL cells both *in vitro* and in an *in vivo* mouse model	(24)
	In migrating CLL cells, the FAK signaling pathway is activated and FAK inhibition decreased CLL cell migration/invasion capabilities	(25)
	FAK is activated in CLL cells after BCR stimulation in a calpain-dependent manner, especially in poor prognosis UM-IGHV patients	(26)
	BCR-dependent FAK activation can be impaired using multi-kinase inhibitors	(29)
**Acute myeloid leukemia (AML)**	FAK expression in AML cells associated with poor prognosis	(33, 34)
	RUNX1 mutations in some high-risk AML patients help in explaining the high FAK expression in these cases with a mechanism involving two negative FAK regulators, i.e., miRNAs hsa-let7a-2-3p and hsa-miR-135a-5p	(35)
	The presence of some FAK splicing isoforms is associated with poor prognosis in AML	(99)
	Contemporary FAK and BCL-2 inhibition had a synergistic anti-leukemic effect in AML, both *in vitro* and in an *in vivo* mouse model	(37)
	FAK inhibition and gene silencing reduced AML cell migration and adhesion to bone marrow mesenchymal stromal cells *in vitro*; FAK inhibition had anti-leukemia effects in an AML mouse model	(34)
**T-cell acute lymphoblastic leukemia (T-ALL)**	Integrin VLA-4 and FAK homologous protein PYK2 were in part responsible for the doxorubicin-based chemotherapy resistance of T-ALL cells	(44)
	Targeting of FAK in an *in vivo* PTEN null T-ALL mouse model increased the anti-leukemia efficacy of PI-3K inhibitors	(45)
**B-cell acute lymphoblastic leukemia (B-ALL)**	FAK gene silencing combined with mTOR inhibition with rapamycin had important anti-leukemia effects both *in vitro* and in an *in vivo* mouse model	(46)
	FAK inhibition had anti-leukemia properties in Ph^+^ ALL and increased the cytotoxic effects of BCR-ABL1 inhibitors, such as dasatinib and nilotinib, in an *in vivo* mouse model	(47, 48)
	FAK gene silencing had anti-cancer properties in Ph^+^ ALL and sensitized leukemic cells to the BCR-ABL1 inhibitor imatinib both *in vitro* and in an *in vivo* mouse model	(49)
**Mantle cell lymphoma (MCL)**	FAK was upregulated/activated in MCL cells in a CXCL12-dependent manner, FAK inhibition and downmodulation decreased the migration capabilities of MCL cells, and FAK inhibition was capable of overcoming Ibrutinib-resistance in MCL	(56)
	SOX11 positivity is typically associated with aggressive MCL due to FAK upregulation and the consequent activation of survival and proliferation pathways, such as AKT and ERK1/2, in lymphoma cells	(57)
	Hedgehog inhibition decreased FAK and integrin VLA-4 expression in MCL cells and, as consequence, their adhesion and migration capabilities	(58)
**Diffuse large B-cell lymphoma (DLBCL)**	High FAK expression was a marker of a good prognosis in DLBCL	(73)
	FAK expression levels in DLBCL cells influenced their response to the treatment with the pan focal adhesion proteins inhibitor E7123	(74)
**Non-Hodgkin lymphoma (NHL) in general**	A higher FAK expression in endothelial cells promotes NF-kB-dependent cytokine production, which provides support to lymphoma cells against DNA-damaging therapies	(76)
**Multiple myeloma (MM)**	FAK expression and activation was higher in MM patients with advanced disease and extramedullary infiltration	(82)
	A decrease in FAK expression and activation is at the base of the anticancer mechanism of action of asiatic acid in MM	(83)
	Inhibition or gene silencing of the FAK homologous protein PYK2 reduced proliferation and adhesion and promoted apoptosis in MM cells both *in vitro* and in an *in vivo* mouse model, also overcoming the support to MM cells coming from the microenvironment	(84, 85)
	FAK inhibition overcame hypoxia-mediated resistance to bortezomib in MM cells and it also increased the anti-cancer effects of bortezomib on MM cells	(86)
**Myelodysplastic syndromes (MDS)**	Mesenchymal stromal cells (MSCs) from bone marrow of low-risk MDS patients were FAK deficient. This finding was associated with morphological and functional changes of MSCs and to an altered hematopoiesis, in particular with an impaired erythroid differentiation.	(98)

In each of the herein faced hematological malignancies, FAK seems to be involved in the pathological behavior of blood tumor cells; FAK also appears to have an important effect on the TME. Beyond leukemias and lymphomas, there is some evidence that extends the importance of FAK in the hematological context globally. For instance, in myeloproliferative neoplasm (MPN, i.e., polycythemia vera, essential thrombocythemia, and primary myelofibrosis) cell lines (100), the inhibition of FAK with PF 562271 (5–10 µM) induced an increase in apoptosis (101).

The implication of FAK in emerging topics (e.g., miRNA and microvesicle fields) has not yet been thoroughly studied, at least in the field of onco-hematological diseases. We reported that, in AML, hsa-let7a-2-3p and hsa-miR-135a-5p miRNA regulated *PTK2*, the gene that encodes the FAK protein (35). The correlation of FAK with other miRNAs has been demonstrated in other pathologies other than onco-hematological ones and these data could provide interesting ideas for applications in lymphoproliferative diseases. As an example, in osteosarcoma, miR-133b decreased the expression of the predicted target genes *BCL2L2*, *MCL-1*, and *IGF1R*, as well as the expression of phospho-Akt and FAK (102). Of note, miR-133b is downregulated in CLL (103). A miRNA downregulated in AML (104), namely miR-485-5p, is involved in a signaling cascade comprising FAK/Src/ERK, thus influencing the biological functions in pancreatic cancer cells (105). Furthermore, it has been demonstrated that miR-373 can inhibit cell proliferation in colon cancer by regulating the FAK-PI3K-AKT and MAPK pathways (106), and the downregulation of miR-373 was confirmed in childhood B-cell precursor ALL (107). These and other examples make clear that the study of the FAK-miRNA relationship could also be interesting in hematological diseases.

In another field, using a mouse embryonic fibroblast cell line induced to express an oncogenic form of diffuse B-cell lymphoma, Kreger and colleagues demonstrated how the oncogenic transformation influenced the biogenesis of extracellular vesicles containing a unique signaling protein that was precisely FAK protein, thus helping in propagating the transformed phenotype (108).

Most chronic lymphoproliferative diseases remain incurable. FAK may represent a player deserving of investigation in an attempt to further dissect the abnormal signaling network in leukemic cells and the inhibition of pro-survival signals derived from the microenvironment. Studies on the promising potential of FAK as a therapeutic target have herein been reviewed.

FAK has emerged as an attractive target for the development of anticancer drugs, and small molecule inhibitors of FAK have demonstrated promising activity. Wang et al. compiled an overview of globally approved FAK drugs for clinical or preclinical trials (109), none of which has yet received marketing authorization. Even worse, none of these trials have been conducted on onco-hematological diseases. Nevertheless, in the first months of this year, several authors have published reviews on the potential of FAK as a therapeutic target, indicating that this topic is particularly relevant (110–112). Among other types of molecules, FAK degraders through proteolysis-targeting chimera (PROTAC) technology have been produced. These compounds can eliminate both kinase-dependent activity and scaffold function by inducing the degradation of FAK (113). PROTACs represent an emerging strategy in the development of novel therapies and some of them have already been developed for research on hematological diseases (i.e., BTK PROTAC or BCL-2 PROTAC). Although new biological drugs have dramatically changed the life expectations of patients with onco-hematological diseases, an increasing number of patients will progressively become refractory to those agents and FAK inhibitors could represent a weapon to overcome the acquired resistance to new inhibitors. For the same reason, drug combinations containing a FAK inhibitor should also be exploited in the onco-hematological field. Current research indicates, in fact, that FAK inhibitors show promising results when combined with other drugs.

In the light of all this evidence, future 360 degree studies on the role of FAK in onco-hematological diseases deserve to be carried out to fill the knowledge gap related to this protein in the different contexts of blood tumors, with the final goal of finding a new appealing target for treatment.
